# Predictive Value of the ALBI Grade for Short-Term Morbidity and Mortality After Pancreatoduodenectomy

**DOI:** 10.3390/cancers18172897

**Published:** 2026-09-07

**Authors:** Ali Ramouz, Abdulelah Al-Ahdal, Carl-Stephan Leonhardt, Georgios Polychronidis, Thomas Hank, Arianeb Mehrabi, Oliver Strobel, Martin Loos, Markus W. Büchler, Zoltan Czigany, Mohammed Al-Saeedi

**Affiliations:** 1 Department of General, Visceral and Transplantation Surgery, Heidelberg University Hospital, 69120 Heidelberg, Germany; 2 Department of General Surgery, Division of Visceral Surgery, Medical University of Vienna, 1090 Vienna, Austria; 3 Botton-Champalimaud Pancreatic Cancer Centre, Champalimaud Foundation, 1400-038 Lisbon, Portugal; 4 Department of General Surgery, Hamad General Hospital, Doha P.O. Box 3050, Qatar

**Keywords:** hyperbilirubinemia, hypoalbuminemia, ALBI, pancreatoduodenectomy, pancreatic surgery

## Abstract

**Background**: Pancreatoduodenectomy (PD) is a complex surgical procedure with substantial postoperative morbidity and mortality. Postoperative outcomes following PD are typically determined by measuring preoperative liver function in the form of serum albumin and bilirubin levels. In this study, the prognostic value of the preoperative ALBI grade was compared with that of albumin and bilirubin alone in predicting postoperative complications and short-term mortality after PD. **Methods**: This retrospective cohort study included 1149 patients who underwent partial or total PD between 2014 and 2017 at a high-volume surgical center. The preoperative ALBI grade (I–III) was calculated in these patients as well as the preoperative levels of bilirubin and albumin. The associations between ALBI grade, albumin and bilirubin levels with postoperative complications and 30- and 90-day mortality were analyzed using univariate and multivariable logistic regression models. The predictive performance of the ALBI grade and albumin and bilirubin levels was assessed using receiver operating characteristic (ROC) curves and area under the curve (AUC) analysis. **Results**: Higher ALBI grades were significantly associated with worse perioperative outcomes, including increased incidence of liver perfusion failure (42.4%) and postpancreatectomy hemorrhage (15.2%). In a pre-specified reduced multivariable model adjusted for BMI, ASA physical status as well as surgical complexity, ALBI grade III remained associated with 30- and 90-day mortality (OR 6.21; 95% CI, 1.62–23.78; *p* = 0.008 and OR 8.97; 95% CI, 2.91–27.62; *p* < 0.001) but was attenuated compared to univariate analyses. Seven of the 8 patients with ASA grade IV physical status were in ALBI grade III and accounted for 5 of the 6 30-day and 6 of the 8 90-day deaths in this group; in a sensitivity analysis excluding these patients, the association between ALBI grade III and 30-day mortality was no longer statistically significant, and the univariable association with 90-day mortality was also attenuated to non-significance, although the BMI-adjusted 90-day estimate remained significant (OR 5.02; 95% CI, 1.07–23.54; *p* = 0.041). ROC analysis showed that the ALBI score (AUC, 0.74 and 0.74) did not outperform albumin alone (AUC, 0.69 and 0.70) in predicting 30- and 90-day mortality (DeLong *p* = 0.21–0.24), although both outperformed bilirubin alone (AUC, 0.54–0.56). **Conclusions**: Preoperative ALBI grade III is associated with short-term morbidity and mortality after PD, but this association is confounded in part by baseline surgical risk (ASA physical status), and the ALBI score does not outperform albumin alone as a predictor of mortality. As a simple, readily available composite marker, the ALBI grade may still help flag patients who warrant closer perioperative surveillance, although this retrospective study does not establish that correcting albumin or bilirubin levels before surgery improves outcomes.

## Simple Summary

Removal of part of the pancreas and small intestine, known as a Whipple procedure, treats pancreatic and nearby cancers but carries a real risk of death and complications in the weeks afterward. This study examined whether a simple blood test score, combining bilirubin and albumin levels, could help identify patients at highest risk before surgery. In a group of over 1100 patients undergoing this operation, those with the worst score had a substantially higher chance of dying within 30 and 90 days after surgery, and spent longer in intensive care and in hospital, than patients with better scores. Part of this risk was explained by overall health and the difficulty of the operation, and whether the score adds useful information beyond these factors was not directly tested in this study. These findings suggest the score could help surgical teams identify higher-risk patients beforehand, allowing closer monitoring and clearer conversations between doctors and patients about the risks of surgery.

## 1. Introduction

Pancreatoduodenectomy (PD) is a challenging surgical procedure [1,2]. Despite recent advances in surgical techniques and postoperative care, morbidity following PD is still high [3,4,5]. Mortality can be predicted by several perioperative variables, which have been associated with complications after PD. These variables include age, male gender, obesity, preoperative hyperbilirubinemia, pancreatic texture, and the extent of resection [6,7]. While most of these factors cannot be modified, liver function can be tested, and hyperbilirubinemia can be addressed with preoperative biliary drainage; however, evidence that this reduces the number and severity of complications after PD remains mixed [8,9,10].

The albumin-bilirubin (ALBI) grade is calculated using serum albumin and bilirubin levels. It was initially developed to assess liver function in patients with hepatocellular carcinoma [11], but has since been recognized as a valuable prognostic tool in patients undergoing pancreatic surgery, particularly cancer-related surgeries [12,13,14]. Patients with higher ALBI grades have significantly shorter overall survival than those with low ALBI grades [13,14]. The ALBI grade can also independently predict the prognosis of these patients [13,14]. However, the association between the ALBI grade and short-term outcomes following PD has not been well studied.

In this study, the associations between the preoperative ALBI grade as well as individual albumin and bilirubin levels and short-term outcomes after PD were assessed.

## 2. Materials and Methods

### 2.1. Study Design and Patient Cohort

All patients who underwent partial or total PD between January 2014 and December 2017 at the Department of General, Visceral and Transplantation Surgery at Heidelberg University Hospital were considered for inclusion in the analysis. The clinical data of included patients were taken from a prospectively maintained institutional database of patients undergoing pancreatic surgeries. Ethical approval was obtained from the institutional ethics committee (S-011/2015). This study was conducted and reported in accordance with the Strengthening the Reporting of Observational Studies in Epidemiology (STROBE) guidelines [15].

### 2.2. Preoperative Data

Clinical data encompassed patient demographics (age, gender, BMI), ASA physical status classification, and preoperative albumin and bilirubin levels. Patients were stratified into three groups according to their bilirubin levels. These groups were mild hyperbilirubinemia (<10 mg/dL), moderate hyperbilirubinemia (10–15 mg/dL), or severe hyperbilirubinemia (>15 mg/dL). Patients were also stratified into three groups according to their serum albumin levels. These groups were no hypoalbuminemia (>3.5 g/dL), mild hypoalbuminemia (3.0–3.5 g/dL), or moderate/severe hypoalbuminemia (<3.0 g/dL) [16]. The ALBI score was also calculated from albumin and bilirubin values to assess preoperative liver function using the following formula [11]:

ALBI score = (log_10_ bilirubin in µmol/L × 0.66) + (albumin in g/L × −0.085)

with bilirubin converted to µmol/L (1 mg/dL = 17.1 µmol/L) and albumin converted to g/L (1 g/dL = 10 g/L). Based on the resulting score, patients were classified into three ALBI grades: grade I (≤−2.60), grade II (>−2.60 to ≤−1.39), or grade III (>−1.39), with increasing ALBI grades corresponding to increasing severity of hepatic dysfunction [11].

Preoperative interventions such as biliary stenting, percutaneous transhepatic cholangiodrainage, and administration of neoadjuvant chemotherapy or radiotherapy were also recorded. Surgical parameters included the surgical indication, type of pancreatic resection, and whether extended or vascular resections were carried out, classified in accordance with the International Study Group on Pancreatic Surgery (ISGPS) [17].

### 2.3. Intraoperative and Postoperative Data

Intraoperative data comprised estimated blood loss and operation duration. The difficulty of the PD procedure was graded according to the classification introduced by Mihaljevic et al., as standard procedures, additional venous resection, multivisceral resection, and additional arterial resection [17]. Postoperative data included postoperative complications, which were evaluated using the criteria set by the ISGPS and categorized using criteria set by the International Study Group on Liver Surgery. These complications included postoperative pancreatic fistula (POPF) [3], postpancreatectomy hemorrhage (PPH) [18], postpancreatectomy bile leakage [19], and liver perfusion failure (LPF). LPF was graded as mild, moderate, or severe based on the classification by Hackert et al. [20]. Postoperative data also included the lengths of stay in the intensive care unit (ICU) and intermediate care unit (IMCU) and the total duration of hospitalization. Mortality was reported as all-cause death occurring within 30- and 90 days after PD.

### 2.4. Statistical Analysis

Statistical analysis was conducted using IBM SPSS Statistics version 29.0 (IBM Corp., 2024, Armonk, NY, USA). Continuous variables were expressed as medians with interquartile ranges (IQR), while categorical variables were presented as counts and percentages. Factors affecting 30- and 90-day mortality were identified by univariate analyses, which involved the Kruskal–Wallis test for continuous variables and either the Chi-square or Fisher’s exact test for categorical variables. In these univariate analyses, variables with a *p* value under 0.1 were included in a multivariable binary logistic regression model to assess independent predictors of 30- and 90-day mortality. Observations with missing data were excluded from the multivariable analysis. The outcomes were presented as odds ratios (ORs) with 95% confidence intervals (CIs). Bonferroni correction was used to adjust for multiple comparisons. Given the limited number of mortality events (25 at 30 days, 36 at 90 days) relative to the number of candidate variables, we defined a priori a reduced multivariable model containing ALBI grade, ASA physical status, and BMI, and surgical complexity, as the primary adjusted analyses. The full model containing all candidate variables is reported as an exploratory analysis. Furthermore, surgical indication (malignant vs. benign/other) was also tested as a covariate in the reduced model but was not retained, as it was not significantly associated with mortality and did not confound the ALBI-mortality association. We additionally performed a sensitivity analysis excluding the 8 patients with ASA grade IV physical status. To evaluate whether the fixed-weight ALBI formula adds discriminative value beyond its two components, we fitted an additional logistic regression model with albumin and bilirubin entered as separate continuous covariates and compared its AUC with that of the ALBI score using DeLong’s test and a likelihood-ratio test. Cause of death within 90 days of surgery was determined by review of clinical records and, where available, linked to a documented local surgical complication (e.g., hemorrhage, sepsis of abdominal origin, or organ failure secondary to a recorded complication). Among patients with moderate or severe preoperative hyperbilirubinemia, 30- and 90-day mortality was compared descriptively between those who did and did not undergo preoperative biliary drainage (ERCP or PTCD). To assess whether the association between preoperative albumin status and length of ICU/IMCU and hospital stay was mediated by major postoperative complications, hospital stay was additionally compared across preoperative albumin categories restricted to patients who did not experience a major complication (clinically relevant POPF, PPH, bile leak, or moderate/severe LPF), using the Kruskal–Wallis test.

The ability of preoperative albumin, bilirubin, and the ALBI grade to predict 30-day and 90-day mortality following PD was evaluated using ROC curve analyses. The area under the ROC curve (AUC), also known as the c-statistic, was calculated for each variable in relation to 30-day and 90-day mortality. ROC curves were generated using the pROC package in R (version 4.5.0). For each predictor, AUC values were computed along with 95% CIs. AUCs were compared between predictors using DeLong’s test. AUC values range from 0.5 (no discrimination) to 1.0 (perfect discrimination), and values ≥ 0.7 are considered acceptable [21]. In all analyses, the binary outcome was defined as 1 for mortality (case) and 0 for survival (control), and ROC directionality was set so that higher predictor values corresponded to a higher risk of mortality. The Hosmer–Lemeshow chi-square goodness-of-fit test was applied to test model suitability. The DeLong test was performed using the roc.test function in the pROC package in R (R Core Team (2025). R: A language and environment for statistical computing. R Foundation for Statistical Computing, Vienna, Austria. https://www.R-project.org/). *p* values of <0.05 indicated statistical significance.

## 3. Results

### 3.1. Study Cohort

A total of 1429 patients underwent either partial or total PD in our center for different indications between January 2014 and December 2017. Of these, 280 patients (19.6%) were excluded because of data loss and lack of follow-up, leaving 1149 patients whose data were included in the analyses. Of the 1149 included patients, 74.8% underwent partial PD and 25.2% underwent total PD. Demographic and intraoperative data of patients are presented in Tables S1–S3.

### 3.2. Preoperative Bilirubin and Postoperative Outcomes

The associations between postoperative outcomes and preoperative serum bilirubin levels are summarized in Table 1. The incidence of moderate or severe LPF was lower in patients with mild hyperbilirubinemia than in patients with moderate or severe hyperbilirubinemia (9.1% vs. 25%; *p* < 0.001). The rate of 30-day mortality was higher in patients with moderate hyperbilirubinemia than in patients with normal bilirubin levels or mild hyperbilirubinemia (7.8% vs. 1.9%; *p* = 0.005). However, bilirubin levels did not significantly affect the need for postoperative interventions, and the length of ICU or hospital stay.

### 3.3. Preoperative Albumin and Postoperative Outcomes

Patients with moderate/severe hypoalbuminemia had significantly higher rates of clinically relevant PPH (18.7% vs. 6.3%; *p* = 0.022) and LPF (31.3% vs. 9.3%; *p* < 0.001) compared with patients with normal albumin levels. They also more frequently required postoperative interventions (31.3% vs. 16.7%; *p* = 0.033). Short-term mortality was significantly higher in patients with moderate/severe hypoalbuminemia, with increased 30-day mortality (18.8% vs. 1.8%; *p* < 0.001) and 90-day mortality (25.0% vs. 2.5%; *p* < 0.001). In addition, ICU/IMCU and overall hospital length of stay were significantly prolonged in patients with preoperative hypoalbuminemia (*p* < 0.001).

### 3.4. ALBI Grade and Postoperative Outcomes

The associations between postoperative outcomes and ALBI grades are presented in Table 2. Overall, 819 (71.3%), 297 (25.8%) and 33 (2.9%) patients had ALBI grades I, II and III, respectively. Higher rates of clinically relevant PPH were observed in patients with higher ALBI grades, but this association was not significant (15.2% in patients with ALBI grade III vs. 6.1% in patients with ALBI grade I; *p* = 0.107). The rate of moderate/severe LPF was significantly higher in patients with an ALBI grade of III than in patients with an ALBI grade of I (42.4% vs. 8.2%; *p* < 0.001). Mortality was also influenced by the ALBI grade. The rate of both 30- and 90-day mortality was significantly higher in patients with ALBI grade III than in patients with ALBI grade I and II (30-day mortality: 18.2% vs. 1.3% and 2.7%; *p* < 0.001; 90-day mortality: 24.2% vs. 2.0%; *p* < 0.001). Cause-of-death analysis in patients who died within 90 days (n = 36) identified sepsis (13/36, 36.1%), PPH (6/36, 16.7%), and hepatic causes (liver failure/necrosis/perfusion failure; 8/36, 22.2%) as the leading contributors; 29 of the 36 deaths (80.6%) occurred in patients with a documented major local surgical complication (clinically relevant POPF grade B/C, PPH grade B/C, biliary leak, or post-pancreatectomy acute pancreatitis/LPF). Furthermore, the lengths of ICU/IMCU and hospital stays were significantly longer in patients with ALBI grades II than in patients with ALBI grade I (*p* < 0.001 for both).

### 3.5. Predictors of Postoperative Mortality

Univariate analyses revealed several perioperative and patient-related variables that were associated with higher rates of 30- and 90-day mortality (Table 3), including ASA physical status (per grade increase; 30-day: OR 7.37, 95% CI 3.15–17.27, *p* < 0.001; 90-day: OR 4.53, 95% CI 2.31–8.86, *p* < 0.001). ASA physical status was distributed unevenly across ALBI grades: 7 of the 8 patients with ASA grade IV fell within ALBI grade III (n = 33), and none were in ALBI grade I. Reflecting this overlap, 5 of the 6 30-day deaths (83%) and 6 of the 8 90-day deaths (75%) among ALBI grade III patients occurred in patients who also had ASA grade IV physical status. Preoperative T-drainage and PTCD were likewise markedly more frequent in ALBI grade III (21.2% each) than in ALBI grade I (0.1–0.9%). ALBI grade III showed the strongest univariate association with mortality (30-day: OR 16.32; 95% CI, 5.62–47.40; *p* < 0.001; 90-day: OR 16.04; 95% CI, 6.28–40.96; *p* < 0.001), but this association attenuated once other covariates were added to the multivariable models. In the full exploratory model containing all candidate variables (Table 3), ALBI grade III was no longer significantly associated with 30-day mortality (OR 4.24; 95% CI, 0.86–21.02; *p* = 0.077) but remained significantly associated with 90-day mortality (OR 7.61; 95% CI, 1.92–30.20; *p* = 0.004). In this large model, surgical complexity was not independently significant, likely reflecting instability from the large number of candidate variables relative to the number of events, and these estimates should be interpreted with caution. Given the limited number of mortality events (25 at 30 days, 36 at 90 days) relative to the number of candidate variables in this full model (2–3 events per predictor), we defined a model containing only ALBI grade, ASA physical status, BMI, and surgical complexity (Table 4); the larger model above is reported as an exploratory analysis only. In this primary reduced model, ALBI grade III remained significantly associated with both 30-day (OR 6.21; 95% CI, 1.62–23.78; *p* = 0.008) and 90-day mortality (OR 8.97; 95% CI, 2.92–27.62; *p* < 0.001) and surgical complexity was itself an independent predictor of both 30- and 90-day mortality (OR 1.66 and 1.59 per grade, respectively; both *p* < 0.02). Higher BMI also remained independently associated with mortality in this model (30-day: OR 1.11; 95% CI, 1.03–1.19; *p* = 0.004; 90-day: OR 1.12; 95% CI, 1.06–1.20; *p* < 0.001). In a sensitivity analysis excluding the 8 patients with ASA grade IV, the univariable association between ALBI grade III and 30-day mortality was no longer significant (OR 2.94; 95% CI, 0.37–23.65; *p* = 0.311; only 1 death remained among the 26 ALBI grade III patients without ASA IV) and remained non-significant after adjustment for BMI (OR 3.63; 95% CI, 0.44–29.82; *p* = 0.230). The univariable association with 90-day mortality was attenuated (OR 4.18; 95% CI, 0.91–19.19; *p* = 0.066; only 2 deaths remained in this group) and reached borderline significance after adjustment for BMI (OR 5.02; 95% CI, 1.07–23.54; *p* = 0.041) (Table S4). Taken together, these findings indicate that the association between ALBI grade III and short-term mortality is confounded in part, but not entirely, by baseline surgical risk (ASA physical status) rather than reflecting an independent effect of hepatic dysfunction alone. Other variables identified as predictors of mortality in univariate analyses but that did not remain independently significant in the primary reduced model included preoperative biliary drainage (T-drainage and PTCD), total versus partial PD, venous resection, and intraoperative blood loss.

### 3.6. Accuracy of Preoperative Parameters in Predicting Postoperative Mortality

**Figure 1:**
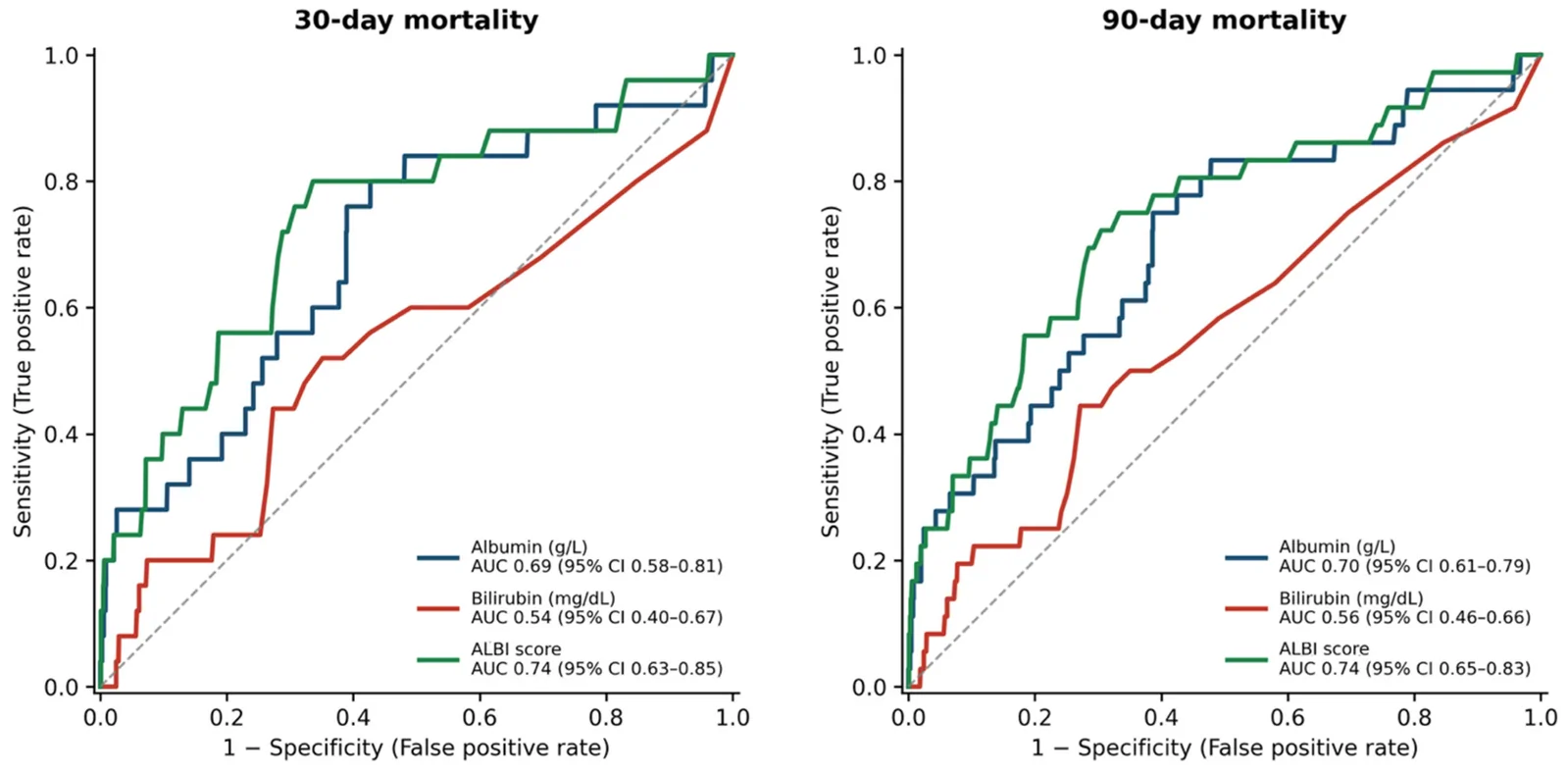
Receiver operating characteristic (ROC) curves for the performance of the ALBI, albumin, and bilirubin scores in predicting 30- and 90-day mortality following pancreatoduodenectomy.

For 30-day mortality, ALBI score had the numerically highest discriminative accuracy (AUC, 0.740; 95% CI, 0.629–0.851), followed by the albumin (AUC, 0.692; 95% CI, 0.578–0.807) and bilirubin alone (AUC, 0.536; 95% CI, 0.403–0.669) (Figure 1). DeLong’s test showed no significant difference between the AUC of the ALBI score and albumin (*p* = 0.240), while ALBI score significantly outperformed bilirubin (*p* = 0.009); the albumin also significantly outperformed bilirubin alone (*p* < 0.001). For 90-day mortality, the pattern was similar: ALBI Score AUC 0.737 (95% CI, 0.647–0.828), albumin AUC 0.699 (95% CI, 0.605–0.793), and bilirubin AUC 0.560 (95% CI, 0.457–0.664), with no significant difference between ALBI and albumin (*p* = 0.205), a significant difference between ALBI score and bilirubin (*p* = 0.003), and a significant difference between the albumin and bilirubin (*p* < 0.001). We also fitted a logistic model with albumin and bilirubin entered as separate continuous covariates; this combined model achieved AUC 0.740 (30-day) and 0.738 (90-day), numerically similar to the fixed-weight ALBI score and the difference was not statistically significant (DeLong *p* = 0.25 and *p* = 0.17, respectively), and a likelihood-ratio test confirmed that bilirubin added no significant discriminative information beyond albumin alone (*p* = 0.95 and *p* = 0.89).

## 4. Discussion

To the best of our knowledge, this is the first study to evaluate the association between preoperative liver function (measured using the ALBI grade, preoperative serum bilirubin, and preoperative serum albumin) and short-term outcomes in a large cohort of patients undergoing PD. Our findings revealed that higher ALBI grade was associated with higher incidence of PPH and postoperative LPF, and, in a pre-specified reduced multivariable model, with both 30- and 90-day mortality; however, the ALBI grade did not outperform albumin alone in predicting short-term mortality.

Hyperbilirubinemia and hypoalbuminemia are both hallmark features of liver dysfunction and are closely interrelated due to the liver’s central role in bilirubin metabolism and albumin synthesis [22,23]. Under proinflammatory conditions, impaired hepatocellular function leads to decreased albumin synthesis and reduced conjugation and excretion of bilirubin [24,25]. Hypoalbuminemia also reduces plasma binding to bilirubin, which increases the proportion of unbound bilirubin, inducing toxicity and impairing clinical outcomes [26,27]. Thus, hyperbilirubinemia and hypoalbuminemia are tightly linked, particularly in the context of hepatic dysfunction and critical illness [22,28]. Other studies have also shown an association between hyperbilirubinemia/hypoalbuminemia and poor outcomes after pancreatic surgery [8,29,30].

In the present study, the rates of POPF, LPF and 30-day mortality were higher in patients with moderate and severe preoperative hyperbilirubinemia than in patients with normal bilirubin levels or mild hyperbilirubinemia. Preoperative hyperbilirubinemia worsens the outcomes of pancreatic surgery primarily because of cholestasis and severe jaundice, which induce a cascade of pathophysiological changes that increase perioperative risk [31,32,33]. Hyperbilirubinemia also impairs hepatic synthetic function, which leads to coagulopathy and a proinflammatory state that promotes infection and impairs wound healing [34]. Although the rate of 30-day mortality was higher in patients with preoperative hyperbilirubinemia, the rate of 90-day mortality was comparable to patients without hyperbilirubinemia. This is in contrast to previous studies in large patient cohorts showing higher rates of 90-day mortality in patients with higher preoperative bilirubin levels (particularly above thresholds of 13 mg/dL or 222 μmol/L) after pancreatic surgery [8].

The present study showed that short-term mortality and postoperative complications (POPF, PPH, biliary leakage, and LPF) following PD were higher in patients who had moderate/severe hypoalbuminemia before surgery than in patients with normal albumin levels before surgery. In agreement with our findings, multiple large cohort studies have demonstrated that preoperative hypoalbuminemia is an independent risk factor for increased postoperative morbidity following PD [30,35,36]. In a recent analysis of over 25,000 patients undergoing PD, hypoalbuminemia (defined as serum albumin < 3.0 g/dL) was independently associated with a significantly higher risk of serious complications in the 30 days following surgery [30]. However, there is currently no evidence to show that correcting hypoalbuminemia with albumin infusion alone can improve surgical outcomes by itself; instead, a more comprehensive preoperative optimization is needed that tackles both albumin and bilirubin levels [30].

The ALBI score, based on preoperative albumin and bilirubin levels, is commonly used in liver surgery as a predictive marker of postoperative complications and for stratification into ALBI grades. A higher ALBI grade and grade III in particular, was associated with increased rates of complications and 30- and 90-day mortality following PD. This association was attenuated, but remained statistically significant at both time points, in our pre-specified primary reduced model adjusted for ASA physical status, BMI, and surgical complexity; however, in a sensitivity analysis excluding the small number of ASA grade IV patients, who accounted for 5 of the 6 30-day and 6 of the 8 90-day deaths among ALBI grade III patients, the association with 30-day mortality was no longer significant. In our corrected ROC analysis, the ALBI grade did not outperform albumin alone in predicting mortality, although it did significantly outperform bilirubin alone. A logistic model combining albumin and bilirubin as separate continuous covariates performed essentially the same as the fixed-weight ALBI score (AUC 0.740 vs. 0.740 at 30 days and 0.738 vs. 0.737 at 90 days), and a likelihood-ratio test showed that bilirubin added no significant discriminative information beyond albumin alone; taken together, these findings indicate that the fixed ALBI formula offers no advantage over modelling albumin and bilirubin directly, and that in this cohort most of the predictive signal for short-term mortality derives from albumin rather than bilirubin. Prior studies have reported superior predictive performance of the ALBI grade compared with other liver function scores, such as MELD-Na, for postoperative morbidity and mortality across patients undergoing liver resections [12,37,38]. Our data suggest that this reflects, at least in part, an overlap between ALBI grade III and baseline surgical risk rather than a purely liver-specific effect. The mechanism underlying the association between ALBI grade and postoperative outcomes is likely multifactorial, reflecting poor nutritional status, increased systemic inflammation, and as our additional analyses suggest, a general marker of reduced physiological reserve that overlaps with but is not identical to ASA physical status. Taken together, our findings indicate that the ALBI grade is a simple, readily available marker that identifies patients at higher perioperative risk, and may be useful clinically to flag such patients for closer perioperative surveillance (e.g., earlier recognition of liver perfusion failure or hemorrhage), individualized discussion of surgical risk versus alternative treatment strategies, and prioritization for future studies of prehabilitation or nutritional optimization; it should not, on the basis of this retrospective association study, be regarded as a robust independent predictor of mortality or as a proven modifiable treatment target.

It is important to note that the ALBI grade reflects hepatic synthetic and excretory function and general physiological reserve rather than pancreatic-specific surgical risk. Postoperative pancreatic fistula, in particular, is predominantly determined by gland texture, duct diameter, and anastomotic technique. Using the classification introduced by Mihaljevic for pancreatoduodenectomy complexity as a proxy for intraoperative difficulty, higher surgical complexity was itself an independent predictor of both 30- and 90-day mortality (OR 1.66 and 1.59 per grade, respectively; both *p* < 0.02) even after adjustment for ALBI grade, ASA physical status, and BMI. Surgical indication (malignant vs. benign/other) was not significantly associated with mortality and did not confound the ALBI-mortality association. The association we observed between ALBI grade and complications such as liver perfusion failure and PPH should therefore not be interpreted as evidence that liver function is a dominant driver of surgical complications broadly, but rather that it identifies a subgroup of physiologically vulnerable patients at higher risk across several complication types. Regarding preoperative biliary stenting, only 24 of 88 patients (27%) with moderate/severe preoperative hyperbilirubinemia in our cohort underwent biliary drainage (ERCP or PTCD), and stented patients did not show lower 30- or 90-day mortality than unstented patients in this small, descriptive, and likely underpowered comparison. Possible explanations include that biliary drainage lowers serum bilirubin biochemically without necessarily reversing the coagulopathy, immune dysfunction, and nutritional depletion associated with prolonged cholestasis in the short interval before surgery. In addition, drainage procedures themselves carry infectious and mechanical complication risk that can offset any benefit and are typically reserved for the most severely jaundiced patients or those awaiting neoadjuvant therapy, so confounding by indication is likely. This is consistent with the mixed evidence in the literature regarding preoperative biliary drainage before pancreatoduodenectomy.

With respect to the association between preoperative hypoalbuminemia and prolonged ICU/IMCU and hospital stay, we tested whether this was simply mediated by major postoperative complications. Among patients who did not experience any major complication (clinically relevant POPF, PPH, bile leak, or moderate/severe LPF), hospital stay was still significantly longer with worsening preoperative albumin status (median 11 days with normal albumin vs. 14.5 days with mild vs. 16 days with moderate/severe hypoalbuminemia; *p* < 0.001). This indicates that the association between hypoalbuminemia and prolonged stay is not fully mediated by the major complications captured in our database, and more likely also reflects reduced physiological and nutritional reserve and slower functional recovery independent of any single complication.

This study has some limitations that should be considered when interpreting the findings. The retrospective observational design introduces the potential for residual confounding despite multivariable adjustment. Data on pancreatic gland texture and specific anastomotic technique were not systematically recorded in our database and could therefore not be included in the multivariable models. Since these are recognized determinants of pancreatic fistula risk, their absence should be considered when interpreting the association between liver-function parameters and postoperative morbidity. Given the limited number of mortality events (25 at 30 days, 36 at 90 days), several multivariable estimates, particularly for sparse categories such as T-drainage, PTCD, and ASA grade IV, are imprecise, as reflected in their wide confidence intervals. The predictive performance of the ALBI grade was primarily assessed using discrimination metrics without formal evaluation of clinical utility. Finally, the ALBI grade was derived from single preoperative laboratory measurements that may be influenced by transient clinical conditions, introducing potential measurement variability.

## 5. Conclusions

The ALBI grade, and grade III in particular, is associated with short-term morbidity and mortality after PD, though this association is confounded in part by baseline patient risk (ASA physical status) and is not superior to albumin alone as a predictor of mortality. As a simple, routinely available composite of two variables that are already measured before surgery, the ALBI grade may be useful for flagging patients who warrant closer perioperative surveillance and multidisciplinary risk discussion. This retrospective association study does not establish that pharmacologically or nutritionally correcting albumin or bilirubin levels before surgery improves outcomes.

## Figures and Tables

**Table 1:** Postoperative data based on the preoperative bilirubin and albumin levels.

	Total	Hyperbilirubinemia			*p* Value	Hypoalbuminemia			*p* Value
	Median (Range) or n (%)	Mild (<10 mg/dL)	Moderate (10–15 mg/dL)	Severe (>15 mg/dL)		None (3.5–5 g/dL)	Mild (3–3.5 g/dL)	Moderate/Severe (2–3 g/dL)	
	*N* = 1149	*N* = 1061	*N* = 64	*N* = 24		*N* = 1075	*N* = 42	*N* = 32	
Clinically relevant POPF *	105 (12.2%)	96 (12.1%)	4 (8.5%)	5 (25.0%)	0.161	98 (12.1%)	4 (12.5%)	3 (18.8%)	0.722
**Clinically relevant PPH**	77 (6.7%)	67 (6.3%)	6 (9.4%)	4 (16.6%)	0.091	68 (6.3%)	3 (7.2%)	6 (18.7%)	**0.022**
**Clinically relevant LPF**	117 (10.4%)	95 (9.1%)	16 (25%)	6 (25%)	**<0.001**	100 (9.3%)	7 (16.7%)	10 (31.3%)	**<0.001**
**Clinically relevant biliary leakage**	57 (4.9%)	55 (5.2%)	1 (1.6%)	1 (4.2%)	0.425	52 (4.8%)	3 (7.1%)	2 (6.3%)	0.751
**Postoperative intervention**	201 (17.5%)	188 (17.7%)	9 (14.1%)	4 (16.7%)	0.752	180 (16.7%)	11 (26.2%)	10 (31.3%)	**0.033**
**30-day mortality**	25 (2.2%)	20 (1.9%)	5 (7.8%)	0 (0.0%)	**0.005**	19 (1.8%)	0 (0.0%)	6 (18.8%)	**<0.001**
**90-day mortality**	36 (3.1%)	30 (2.8%)	5 (7.8%)	1 (4.2%)	0.081	27 (2.5%)	1 (2.4%)	8 (25.0%)	**<0.001**
**ICU/IMCU stay (days)**	1 (1–4)	1 (1–4)	1.5 (1–4)	1 (1–3)	0.650	1 (1–4)	3 (1–8.5)	7 (1–20)	**<0.001**
**Hospital stay (days)**	13 (10–20)	13 (9–20)	13 (10–19)	14 (11–19)	0.630	13 (9–19)	16 (11–23)	19.5 (13–37)	**<0.001**

*p* values in bold indicate statistically significant differences. Values are n (%) or median (range). * Among patients undergone partial pancreatoduodenectomy. Abbreviations: ICU = intensive care unit, IMCU = intermediate care unit, IQR = interquartile range, LPF = liver perfusion failure, PPH = postpancreatectomy hemorrhage.

**Table 2:** Postoperative data with ALBI grade.

	Total	ALBI Grade			*p* Value
	Median (IQR) or n (%)	I	II	III	
	*N* = 1149	*N* = 819	*N* = 297	*N* = 33	
POPF *	105 (12.2%)	72 (11.8%)	31 (13.4%)	2 (10.5%)	0.798
**PPH**	77 (6.7%)	50 (6.1%)	22 (7.4%)	5 (15.2%)	0.107
**Liver perfusion failure**	117 (10.4%)	67 (8.2%)	38 (12.8%)	14 (42.4%)	**<0.001**
**Biliary leakage**	57 (4.9%)	47 (5.7%)	9 (3.0%)	1 (3.0%)	0.160
**Postoperative intervention**	201 (17.5%)	135 (16.5%)	60 (20.2%)	6 (18.2%)	0.350
**30-day mortality**	25 (2.2%)	11 (1.3%)	8 (2.7%)	6 (18.2%)	**<0.001**
**90-day mortality**	36 (3.1%)	16 (2.0%)	12 (4.0%)	8 (24.2%)	**<0.001**
**ICU/IMCU stay (days)**	1 (1–4)	1 (1–4)	3 (1–13.5)	4.5 (1–20)	**<0.001**
**Hospital stay (days)**	13 (10–20)	13 (9–20)	17.5 (12–29.7)	12 (9–27.5)	**<0.001**

*p* values in bold indicate statistically significant differences. * Among patients undergone partial pancreatoduodenectomy. Abbreviations: ALBI = albumin-bilirubin grade, ICU = intensive care unit, IMCU = intermediate care unit, IQR = interquartile range, POPF = postoperative pancreatic fistula, PPH = postpancreatectomy hemorrhage.

**Table 3:** Uni- and multivariate analyses of independent predictors for 30- and 90-day mortality.

	30-Day/90-Day Events	30-Day Mortality						90-Day Mortality					
		Univariate Analysis			Multivariate Analysis			Univariate Analysis			Multivariate Analysis		
		OR	CI	*p*	OR	CI	*p*	OR	CI	*p*	OR	CI	*p*
**Age**	25/36	1.033	0.994–1.073	0.099	1.039	(0.989–1.091)	0.132	1.032	1.000–1.066	0.053	1.040	(0.998–1.084)	0.063
**Gender (female vs. Male)**	17/22	1.584	0.678–3.700	0.288				1.167	0.591–2.306	0.656			
**BMI** (**per kg**/**m^2^)**	25/36	1.127	1.051–1.208	**<0.001**	1.132	1.053–1.217	**<0.001**	1.126	1.062–1.194	**<0.001**	1.125	1.058–1.196	**<0.001**
**ASA physical status (per grade)**	24/34	7.371	3.145–17.274	**<0.001**	3.093	1.267–7.551	0.013	4.527	(2.314–8.855)	**<0.001**	1.867	0.902–3.865	0.093
**Bile duct stent**	7/10	0.98	0.405–2.369	0.964				0.968	0.461–2.030	0.931			
**T-drainage**	2/2	9.687	2.009–46.717	**0.005**	3.534	0.535–23.329	0.190	6.482	1.368–30.727	**0.019**	1.853	0.299–11.476	0.507
**PTCD**	3/4	7.514	2.079–27.153	**0.002**	2.690	0.385–18.797	0.318	7.178	2.309–22.310	**0.001**	2.250	0.365–13.878	0.382
**Neoadjuvant chemotherapy**	2/6	0.727	0.169–3.121	**0.668**				1.716	0.699–4.208	**0.238**			
**Neoadjuvant radiochemotherapy**	4/8	1.162	0.394–3.430	0.786				1.774	0.794–3.963	0.162			
**PD (partial or total)**	11/15	2.391	1.073–5.328	**0.033**	1.162	0.429–3.149	0.768	2.185	1.111–4.297	**0.024**	1.291	0.517–2.871	0.651
**Surgical complexity (per grade)**	25/36	1.693	1.174–2.442	**0.005**	1.477	0.897–2.433	0.125	1.654	(1.215–2.252)	**0.001**	1.320	0.833–2.093	0.237
**Venous resection**	9/15	1.785	0.773–4.124	**0.175**				2.264	1.143–4.482	**0.019**	0.986	0.383–2.539	0.977
**Arterial resection**	1/2	0.971	0.128–7.341	0.977				1.368	0.319–5.873	0.673			
**Blood loss (per mL)**	25/36	1	1.000–1.000	**0.003**	1	1.000–1.000	0.139	1	1.000–1.000	**0.001**	1	1.000–1.000	0.198
**Oeration duration (per min)**	25/36	1.004	1.000–1.008	**0.061**	1.003	0.997–1.008	0.344	1.005	1.002–1.009	**0.004**	1.004	1.000–1.009	0.072
**ALBI I**	11/16	Ref.						Ref.					
**ALBI II**	8/12	2.033	0.810–5.105	0.131	1.465	0.527–4.076	0.464	2.111	0.986–4.516	0.054	1.554	0.654–3.690	0.318
**ALBI III**	6/8	16.323	5.621–47.402	**<0.001**	4.242	0.856–21.017	0.077	16.040	6.282–40.956	**<0.001**	7.613	1.920–30.197	**0.004**

*p* values in bold indicate statistically significant differences. Abbreviations: ALBI = albumin-bilirubin grade, ASA = American Society of Anesthesiology, BMI = body mass index, CI = confidence interval, OP = operation; OR = odds ratio, PD = pancreatoduodenectomy, PTCD = percutaneous transhepatic cholangiodrainage.

**Table 4:** Parsimonious multivariable model (ALBI grade, ASA physical status, BMI, and surgical complexity) for 30- and 90-day mortality after PD.

Variable	30-Day Mortality, OR (95% CI), *p*	90-Day Mortality, OR (95% CI), *p*
**ALBI grade I**	Reference	Reference
**ALBI grade II**	1.690 (0.634–4.507), *p* = 0.294	1.731 (0.760–3.943), *p* = 0.192
**ALBI grade III**	6.211 (1.623–23.776), *p* = **0.008**	8.972 (2.915–27.617), *p* < **0.001**
**ASA (per grade)**	3.231 (1.375–7.590), *p* = **0.007**	2.167 (1.099–4.273), *p* = **0.026**
**BMI (per kg/m^2^)**	1.108 (1.032–1.190), *p* = **0.004**	1.124 (1.057–1.195), *p* < **0.001**
**Surgical complexity of Whipple (Mihaljevic et al., per level)**	1.659 (1.090–2.525), *p* = **0.018**	1.594 (1.117–2.273), *p* = **0.010**

Abbreviations: ASA, American Society of Anesthesiology, BMI, body mass index, OR = odds ratio. Model includes the full cohort (N = 1149). Surgical indication (malignant vs. benign/other, using linked diagnosis data) was tested as an additional covariate but was not significantly associated with mortality (30-day: OR 0.55, 95% CI 0.22–1.38, *p* = 0.20; 90-day: OR 0.78, 95% CI 0.35–1.72, *p* = 0.53) and did not materially change the other estimates, so it was not retained in the final model. *p* values in bold indicate statistically significant differences.

## Data Availability

The data underlying this study are available from the corresponding author upon reasonable request.

## Supplementary Materials

The following supporting information can be downloaded at https://www.mdpi.com/article/10.3390/cancers18172897/s1, Table S1. Demographic data and preoperative bilirubin levels; Table S2. Demographic data and preoperative albumin levels; Table S3. Demographic data and ALBI grade; Table S4. Sensitivity analysis: association between ALBI grade III and mortality after excluding the 8 patients with ASA grade IV.
