# Supplementary material for: Predictive Value of the ALBI Grade for Short-Term Morbidity and Mortality After Pancreatoduodenectomy
Source: Cancers (Basel). 2026 Sep 7;18(17):2897. doi: 10.3390/cancers18172897 (PMC13565257; doi:10.3390/cancers18172897)
Supplement: Supplementary file 1 [file cancers-18-02897-s001.zip › cancers-4460676-supplementary.pdf]

Supplementary Appendix

Predictive Value of The ALBI Grade for Short-Term Morbidity and Mortality After Pancreatoduodenectomy

Ali Ramouz[1], Abdulelah Al-Ahdal[1], Carl-Stephan Leonhardt[1], Georgios Polychronidis[1], Thomas Hank[1], Arianeb Mehrabi[1], Oliver Strobel[1,2], Martin Loos[1], Markus W. Büchler[1,3], Zoltan Czigany[1*], Mohammed Al-Saeedi[1,4*]

[1]Department of General, Visceral and Transplantation Surgery, Heidelberg University Hospital, Heidelberg, Germany

[2]Department of General Surgery, Division of Visceral Surgery, Medical University of Vienna, Vienna, Austria

[3]Botton-Champalimaud Pancreatic Cancer Centre, Champalimaud Foundation, Lisbon, Portugal

[4]Department of General Surgery, Hamad General Hospital, Doha, Qatar

*ZC and MAS contributed equally.

Corresponding author and requests for reprints:

Mohammed Al-Saeedi, MD, MBA

Vice Chairman

Head of Division – Surgical Oncology

General-, Visceral- & Transplantation Surgery

University Hospital Heidelberg,

Im Neuenheimer Feld 420

D-69120 Heidelberg

Mohammed.al-saeedi@med.uni-heidelberg.de

Running title

ALBI predicts outcomes of panc(1)reatoduodenectomy

Study cohort

The median age was 65 years (range, 56–73), and the median BMI was 24.8 kg/m² (range, 22.4–27.7). Of the total cohort, 488/1149 (42.5%) patients were female and 661/1149 (57.5%) were male. Most patients had an ASA physical status classification of II (58.9%), followed by III (37.5%), I (2.9%), and IV (0.7%). The most common indication for partial or total PD was ductal adenocarcinoma (723 patients; 62.9%), followed by intraductal papillary mucinous neoplasia (221 patients; 19.3%), chronic pancreatitis (62 patients; 5.4%), periampullary carcinoma (46 patients; 4.0%), pancreaticoduodenal neuroendocrine neoplasia (32 patients; 2.8%), distal cholangiocarcinoma (28 patients; 2.4%), duodenal adenocarcinoma (24 patients; 2.1%), and gastrointestinal stromal tumors (13 patients; 1.1%). Preoperative biliary drainage was performed in 31.4% of patients; 28.4% of patients had a bile duct stent, 1.0% had a T-drain, and 2.1% underwent percutaneous transhepatic cholangiographic drainage (PTCD). Neoadjuvant treatment was administered to 24.7% of patients; 10.6% received chemotherapy and 14.1% received combined chemoradiotherapy.

Among the 1149 patients who underwent PD, 74.8% underwent partial PD and 25.2% underwent total PD. Extended resections were performed in 19.8% of patients. Venous resection was required in 13.4% of patients, and arterial resection was performed in 4.3% of patients. The median operation time was 325 minutes (range, 275–380 minutes), and the median intraoperative blood loss was 1000 mL (range, 600–1500 mL). POPF were observed in 26.6% of patients, and clinically relevant fistulas (grade B/C) in 12.3% of patients. PPH was noted in 7.9% of patients, and clinically relevant hemorrhage (grade B/C) in 6.7% of patients. LPF was reported in 18.6% of patients, and the combined incidence of moderate to severe liver perfusion failure was 10.4%. Postoperative interventions were required in 17.5% of patients. The 30-day mortality rate was 2.2%, and the 90-day mortality rate was 3.1%.

Table S1. Demographic data and preoperative bilirubin levels.

	Total	Preoperative hyperbilirubinemia			*P* value
	Median (IQR) or n (%)	Mild (< 10 mg/dL)	Moderate (10–15 mg/dL)	Severe (>15 mg/dL)	
	(n = 1149)	(n = 1061)	(n = 64)	(n = 24)	
**Demographic data**					
**Age**	65 (56–73)	65 (56–73)	65 (57–73)	70 (62–73)	0.142
**BMI**	24.8 (22.4–27.7)	24.7 (22.3–27.6)	25.3 (23.2–28.4)	25.1 (22.6–26.9)	0.395
**Gender**					
• Female	488 (42.5%)	452 (42.6%)	28 (43.8%)	8 (33.3%)	0.647
• Male	661 (57.5%)	609 (57.4%)	36 (56.3%)	16 (66.7%)	
**ASA classification**					
• I	33 (2.9%)	32 (3.0%)	1 (1.6%)	0(0.0%)	0.090
• II	677 (58.9%)	624 (58.8%)	39 (61.3%)	14 (57.1%)	
• III	431 (37.5%)	400 (37.7%)	22 (33.9%)	9 (38.1%)	
• IV	8 (0.7%)	5 (0.5%)	2 (3.2%)	1 (4.8%)	
**Preoperative interventions**					
• Bile duct stent	326 (28.4%)	304 (28.7%)	13 (20.3%)	9 (37.5%)	0.215
• T-drainage	12 (1.0%)	8 (0.8%)	3 (4.7%)	1 (4.2%)	**0.003**
• PTCD	23 (2.0%)	18 (1.7%)	2 (3.1%)	3 (12.5%)	**< 0.001**
**Neoadjuvant therapy**					
• Chemotherapy	122 (10.6%)	122 (11.5%)	0 (0.0%)	0 (0.0%)	**0.003**
• Chemoradiotherapy	162 (14.1%)	160 (15.1%)	2 (3.2%)	0 (0.0%)	**0.004**
**Surgical complexity (Mihaljevic et al.)**					
• 0	719 (62.6%)	675 (63.6%)	28 (43.8%)	16 (66.7%)	**0.014**
• 1	154 (13.4%)	133 (12.5%)	16 (25.0%)	5 (20.8%)	
• 2	227 (19.8%)	207 (19.5%)	18 (28.1%)	2 (8.3%)	
• 3	49 (4.3%)	46 (4.3%)	2 (3.1%)	1 (4.2%)	

P values in bold are statistically significant.

Abbreviations: ASA = American Society of Anesthesiologists physical status classification, BMI = body mass index, IQR = interquartile range, PTCD = percutaneous transhepatic cholangiodrainage

Table S2. Demographic data and preoperative albumin levels.

	**Total**	**Hypoalbunemia**			***P***
	Median (IQR) or n (%)	None (3.5–5 g/dL)	Mild (3–3.5 g/dL)	Moderate/severe (2–3 g/dL)	**value**
	N = 1149	*N* = 1075	*N* = 42	*N* = 32	
**Demographic data**					
**Age**	65 (56–73)	65 (56–72)	71 (59–77)	66 (59–73)	**0.033**
**BMI**	24.8 (22.4–27.7)	24.9 (22.4–27.7)	24.6 (22.2–29.3)	24.4 (22.6–27.8)	0.974
**Gender**					
• Female	488 (42.5%)	463 (43.0%)	16 (38.1%)	9 (28.1%)	0.206
• Male	661 (57.5%)	612 (57.0%)	26 (61.9%)	23 (71.9%)	
**ASA classification**					
• I	33 (2.9%)	31 (2.9%)	2 (4.8%)	0(0.0%)	**<0.001**
• II	677 (58.9%)	655 (60.9%)	15 (35.7%)	7 (21.9%)	
• III	431 (37.5%)	387 (36.0%)	26 (61.9%)	18 (56.2%)	
• IV	8 (0.7%)	0 (0.0%)	1 (2.4%)	7 (21.9%)	
**Preoperative interventions**					
• Bile duct stent	326 (28.4%)	292 (27.2%)	21 (50.0%)	13 (40.6%)	**0.002**
• T-drainage	12 (1.0%)	3 (0.3%)	3 (7.1%)	6 (18.8%)	**< 0.001**
• PTCD	23 (2.0%)	16 (1.5%)	2 (4.8%)	6 (18.8%)	**< 0.001**
**Neoadjuvant therapy**					
• Chemotherapy	122 (10.6%)	113 (10.5%)	6 (14.3%)	3 (9.4%)	0.719
• Chemoradiotherapy	162 (14.1%)	149 (13.9%)	8 (19.0%)	5 (15.6%)	0.620
**Surgical complexity (Mihaljevic et al.)**					
• 0	719 (62.6%)	681 (63.3%)	25 (59.5%)	13 (40.6%)	**0.040**
• 1	154 (13.4%)	143 (13.3%)	7 (16.7%)	4 (12.5%)	
• 2	227 (19.8%)	204 (19.0%)	9 (21.4%)	14 (43.8%)	
• 3	49 (4.3%)	47 (4.4%)	1 (2.4%)	1 (3.1%)	

P values in bold are statistically significant.

Abbreviations: ASA = American Society of Anesthesiologists physical status classification, BMI = body mass index, IQR = interquartile range, PTCD = percutaneous transhepatic cholangiodrainage

Table S3. Demographic data and ALBI grade.

	Total	ALBI grade			*P* value
	Median (IQR) or n (%)	I	II	III	
	N =1149	*N* = 819	*N* = 297	*N* = 33	
**Demographic data**					
**Age**	65 (56–73)	64.0 (18–89)	69 (21–87)	67 (50.5–82)	**< 0.001**
**BMI**	24.8 (22.4–27.7)	24.8 (15.2–46.7)	25.0 (17.1–45.5)	24.7 (15.8–41.2)	0.617
**Gender**					
• Female	488 (42.5%)	357 (43.6%)	120 (40.4%)	11 (33.3%)	0.356
• Male	661 (57.5%)	462 (56.4%)	177 (59.6%)	22 (66.7%)	
**ASA classification**					
• I	33 (2.9%)	26 (3.2%)	7 (2.4%)	0 (0.0%)	**< 0.001**
• II	677 (58.9%)	511 (62.4%)	155 (52.2%)	11 (33.3%)	
• III	431 (37.5%)	282 (34.4%)	134 (45.1%)	15 (45.5%)	
• IV	8 (0.7%)	0 (0.0%)	1 (0.3%)	7 (21.2%)	
**Preoperative interventions**					
• Bile duct stent	326 (28.4%)	192 (23.5%)	119 (40.1%)	15 (45.5%)	**0.007**
• T-drainage	12 (1.0%)	1 (0.1%)	4 (1.3%)	7 (21.2%)	**< 0.001**
• PTCD	23 (2.0%)	7 (0.9%)	9 (3.0%)	7 (21.2%)	**< 0.001**
**Neoadjuvant therapy**					
• Chemotherapy	122 (10.6%)	107 (13.1%)	13 (4.4%)	2 (6.1%)	**< 0.001**
• Chemoradiotherapy	162 (14.1%)	136 (16.6%)	22 (7.4%)	4 (12.1%)	**< 0.001**
**Surgical complexity (Mihaljevic et al.)**					
• 0	719 (62.6%)	540 (65.9%)	167 (56.2%)	12 (36.4%)	**< 0.001**
• 1	154 (13.4%)	81 (9.9%)	65 (21.9%)	8 (24.2%)	
• 2	227 (19.8%)	163 (19.9%)	52 (17.5%)	12 (36.4%)	
• 3	49 (4.3%)	35 (4.3%)	13 (4.4%)	1 (3.0%)	

P values in bold are statistically significant.

Abbreviations: ASA = American Society of Anesthesiologists physical status classification, BMI = body mass index, IQR = interquartile range, PTCD = percutaneous transhepatic cholangiodrainage

Table S4. Sensitivity analysis: association between ALBI grade III and mortality after excluding the 8 patients with ASA grade IV.

Analysis (ASA IV patients excluded, n=1141)	30-day mortality, OR (95% CI), P	90-day mortality, OR (95% CI), P
ALBI grade III vs. I, univariable	2.938 (0.365–23.647), P=0.311	4.177 (0.909–19.195), P=0.066
ALBI grade III vs. I, BMI-adjusted	3.629 (0.442–29.822), P=0.230	5.019 (1.070–23.542), P=0.041

Abbreviations: ASA, American Society of Anesthesiology, BMI, body mass index, OR = odds ratio.

Reference category is ALBI grade I. Only 26 ALBI grade III patients remain after excluding ASA IV, with 1 death at 30 days and 2 deaths at 90 days in this group.
